# Impact of lung adenocarcinoma subtypes on survival and timing of brain metastases

**DOI:** 10.3389/fonc.2024.1433505

**Published:** 2024-09-03

**Authors:** Chuyan Zhou, Xiaofang Zhang, Xingyu Yan, Haitao Xie, Hao Tan, Yingqiu Song, Mo Li, Yi Jin, Tianlu Wang

**Affiliations:** ^1^ Department of Radiotherapy, Cancer Hospital of China Medical University, Liaoning Cancer Hospital & Institute, Cancer Hospital of Dalian University of Technology, Shenyang, Liaoning, , China; ^2^ School of Graduate, China Medical University, Shenyang, China; ^3^ Faculty of Medicine, Dalian University of Technology, Dalian, China

**Keywords:** invasive lung adenocarcinoma, brain metastasis, pathological subtype, prognosis, survival

## Abstract

**Purpose:**

Lung cancer is a devastating disease, with brain metastasis being one of the most common distant metastases of lung adenocarcinoma. This study aimed to investigate the prognostic characteristics of individuals with brain metastases originating from invasive lung adenocarcinoma of distinct pathological subtypes, providing a reference for the management of these patients.

**Methods:**

Clinical data from 156 patients with lung adenocarcinoma-derived brain metastases were collected, including age, sex, smoking status, Karnofsky Performance Status scores, pathological subtype, lymph node metastasis, tumor site, treatment mode, T stage, and N stage. Patients were classified into two groups (highly differentiated and poorly differentiated) based on their pathological subtypes. Propensity score matching was used to control for confounding factors. The prognostic value of pathological subtypes was assessed using Kaplan-Meier analysis and Cox proportional hazards regression modeling.

**Results:**

Kaplan-Meier analysis indicated that patients in the moderately to highly differentiated group had better prognoses. Multivariate analysis revealed that being in the poorly differentiated group was a risk factor for poorer prognosis. Thoracic tumor radiation therapy, chemotherapy, and surgery positively influenced the time interval between lung cancer diagnosis and brain metastasis.

**Conclusions:**

The pathological subtypes of lung adenocarcinoma-derived brain metastases are associated with patient prognosis. Patients in the poorly differentiated group have worse prognoses compared to those in the moderately to highly differentiated group. Therefore, patients in the poorly differentiated group may require more frequent follow-ups and aggressive treatment.

## 1 Introduction

Lung cancer is a worldwide population health concern with a mortality rate higher than that of breast, prostate, and colorectal cancers combined (1). In China, lung cancer has the highest incidence and mortality rate among all cancer types (2, 3). Approximately 85% of lung cancer diagnoses are non-small-cell lung carcinomas (NSCLC) (4). Between 10% and 20% of patients with NSCLC develop brain metastases at presentation, and up to 50% of patients will develop brain metastases during the course of the disease (5, 6). Patients with brain metastases have a poor prognosis and a shortened median survival (7). With the continued development of molecular targeted therapies and immunotherapies, patients with lung cancer are living longer and, therefore, are at greater risk for brain metastases. Although the deleterious effects of brain metastases from lung cancer are widely understood, patients with brain metastases are less sensitive to drug therapy, and surgical interventions are limited. The median survival of patients with brain metastases is typically 4–9 months (8). Brain metastasis significantly impacts patient quality of life and has become a serious global social health problem (9–11).

Lung adenocarcinoma has a greater risk of brain metastasis among patients with NSCLC, according to a long-term follow-up in the US SEER database (12). However, the number of studies on prognostic factors for brain metastases in lung adenocarcinoma is limited. Cell type, primary tumor size, and lymph node stage have been associated with the probability of lung adenocarcinoma brain metastasis (13). Pathological subtypes of lung adenocarcinoma play a considerable role in cancer progression. Invasive non-mucinous adenocarcinomas are divided into five types: lepidic, acinar, papillary, micropapillary, and solid (14). Each pathological subtype has unique histological features that impact patient survival and treatment. Numerous studies have found that micropapillary and solid types are associated with a poorer prognosis, whereas the lepidic growth type is associated with a better prognosis (15–17). Therefore, lung adenocarcinomas with micropapillary and solid components are considered high-risk and require more thorough treatments. In contrast, the other subtypes are categorized as low risk.

Considering that NSCLC comprises numerous types that may be affected by various confounding factors, we selected lung adenocarcinoma, which is prone to brain metastases, as the study subject to reduce interference and improve the accuracy of the study. Moreover, to date, the impact of different pathologic subtypes on the prognosis of patients with brain metastases from aggressive lung adenocarcinoma has not been reported. Therefore, we retrospectively analyzed the relationship between pathologic subtypes of lung adenocarcinoma and survival after brain metastasis. In addition, we evaluated the relationship between pathologic subtypes and the time interval between lung cancer diagnosis and brain metastasis (brain metastasis interval). We evaluated 156 patients with brain metastases from invasive lung adenocarcinoma admitted to our hospital to provide a theoretical basis for future treatment approaches.

## 2 Methods

### 2.1 Study subjects

The clinical data of 156 patients with brain metastases from invasive lung adenocarcinoma were assessed. The patients were treated at the Liaoning Provincial Tumor Hospital (2008–2017). The inclusion criteria were as follows: (1) a clear diagnosis of lung adenocarcinoma and the existence of subtype classification according to clinicopathology or cytology; (2) brain metastasis confirmed by magnetic resonance imaging; and (3) age ≥18 years. The exclusion criteria were as follows: (1) no clear pathological diagnosis or secondary lung cancer; (2) primary tumor at other sites; and (3) incomplete clinical data.

### 2.2 Data collection

The relevant patient information was collected, including pathological type, sex, age, smoking status, Karnofsky Performance Status (KPS) score, lymph node metastasis, tumor site, treatment modality, T stage (size and extent of the primary tumor), and N stage (number of affected lymph nodes).

### 2.3 Grouping method

Lung cancer is divided into three grades: grade 1 indicates high differentiation, with predominantly lepidic growth type and a high-grade pattern (solid, micropapillary, or complex glandular) not exceeding 20%; grade 2 indicates moderate differentiation, with acinar or papillary predominance and a high-grade pattern not exceeding 20%; and grade 3 indicates poor differentiation, where the high-grade pattern is ≥20%. Patients were divided into two groups: a moderate- to high-differentiation group and a poor-differentiation group, according to their pathological subtypes.

### 2.4 Follow-up

The date of the patient’s death or last follow-up was used as the cutoff date.

### 2.5 Statistical methods

After propensity score matching (PSM), the patients from the poor-differentiation group (n = 59) and moderate- to high-differentiation group (n = 59) were matched using a 1:1 ratio. The parameters of patient clinicopathological characteristics and the distinct pathological subtypes were compared using the chi-squared test. Multivariate Cox regression analysis was used to determine the prognostic risk factors. The Kaplan–Meier method was applied to the survival curves to calculate the survival rates (0: moderate- to high-differentiation group; 1: poor-differentiation group), and the difference in survival was compared using the log-rank test. Differences were considered statistically significant when P < 0.05. All statistical analyses were performed using the IBM Statistical Package for the Social Sciences (SPSS) version 25.0.

## 3 Results

### 3.1 Patient characteristics

Hundred fifty-six patients who met the inclusion criteria were recruited for this analysis. Of those patients, 89 (57.1%) had moderately to highly differentiated invasive lung adenocarcinoma, and the remaining 67 (42.9%) had poorly differentiated invasive lung adenocarcinoma. More than half (66.7%) of the individuals were younger than 60 years, 79 (50.6%) were female, and the majority (76.2%) underwent chest chemotherapy. In the poor-differentiation group, patients were more likely to be male (P < 0.05). Lymph node involvement (N2–3; P < 0.05) was more severe in the moderate- to high-differentiation group than in the poor-differentiation group (**Table 1**).

**Table 1:** Baseline characteristics of patients with brain metastases.

Characteristic	0 (high and middle differentiation groups)	1 (low differentiation group)	p
n	89	67	
Age(year), n (%)			0.689
<60	61 (39.1%)	43 (27.6%)	
≥60	28 (17.9%)	24 (15.4%)	
Gender, n (%)			0.038
male	37 (23.7%)	40 (25.6%)	
female	52 (33.3%)	27 (17.3%)	
Whether smoke, n (%)			0.844
no	57 (36.5%)	41 (26.3%)	
yes	32 (20.5%)	26 (16.7%)	
KPS score, n (%)			0.380
≥90	43 (27.6%)	38 (24.4%)	
<90	46 (29.5%)	29 (18.6%)	
Lymph node metastasis, n (%)			0.641
no	24 (15.4%)	15 (9.6%)	
yes	65 (41.7%)	52 (33.3%)	
Whether brain metastases have occurred at the time of diagnosis, n (%)			0.487
no	68 (43.6%)	47 (30.1%)	
yes	21 (13.5%)	20 (12.8%)	
Tumor location, n (%)			0.842
central	15 (9.6%)	13 (8.3%)	
peripheral	74 (47.4%)	54 (34.6%)	
T stage, n (%)			0.294
0~2	64 (41%)	42 (26.9%)	
3~4	25 (16%)	25 (16%)	
N stage, n (%)			0.045
0~1	39 (25%)	18 (11.5%)	
2~3	50 (32.1%)	49 (31.4%)	
TNM stage, n (%)			0.547
1~3	48 (30.8%)	32 (20.5%)	
4	41 (26.3%)	35 (22.4%)	
Radiotherapy, n (%)			0.897
no	71 (45.5%)	52 (33.3%)	
yes	18 (11.5%)	15 (9.6%)	
Chemotherapy, n (%)			0.882
no	22 (14.1%)	15 (9.6%)	
yes	67 (42.9%)	52 (33.3%)	
Surgery, n (%)			0.064
no	36 (23.1%)	38 (24.4%)	
yes	53 (34%)	29 (18.6%)	

### 3.2 Survival analysis of overall survival and the brain metastasis interval before PSM

Case subtype was linked to brain metastasis survival in univariate analyses (hazard ratio [HR]: 1.421; 95% confidence interval [CI]: 1.020–1.981; P = 0.038) but not to the brain metastasis interval (HR: 1.190; 95% CI: 0.853–1.660; P = 0.305). The KPS, primary tumor site, T stage, thoracic tumor stage, thoracic tumor chemotherapy, thoracic tumor radiation therapy, and thoracic tumor surgical therapy were related to the brain metastasis interval (P < 0.05). Patients in the poor-differentiation group had a lower survival rate after brain metastasis (HR: 1.421; 95% CI: 1.020-1.981; P = 0.038) than those in the moderate- to high-differentiation group. As shown by the multivariate analysis, a peripheral primary tumor site (HR: 0.516; 95% CI: 0.331–0.802; P = 0.003), chemotherapy (HR: 0.415; 95% CI: 0.276–0.624; P < 0.001), and surgical treatment for thoracic tumors (HR: 0.266; 95% CI: 0.151–0.469; P < 0.001) were positive prognostic factors for the brain metastasis interval (**Tables 2**, **3**).

**Table 2:** Univariate and multivariate Cox regression analyses before propensity score matching to examine the overall survival.

Characteristics	Total(N)	Univariate analysis		Multivariate analysis	
		Hazard ratio (95% CI)	P value	Hazard ratio (95% CI)	P value
pathological subtype	156	1.421 (1.020-1.981)	**0.038**	1.421 (1.020-1.981)	**0.038**
age	156	1.002 (0.709-1.415)	0.993		
gender	156	0.790 (0.568-1.098)	0.161		
Whether smoke	156	1.175 (0.836-1.652)	0.353		
KPS score	156	1.206 (0.870-1.673)	0.260		
Lymph node metastasis	156	1.153 (0.786-1.690)	0.466		
Whether brain metastases have occurred at the time of diagnosis	156	0.765 (0.526-1.112)	0.161		
Tumor location	156	0.825 (0.541-1.256)	0.369		
T stage	156	0.933 (0.656-1.327)	0.701		
N stage	156	1.173 (0.834-1.649)	0.359		
TNM stage	156	0.852 (0.614-1.181)	0.336		
Thoracic tumor radiation	156	0.961 (0.643-1.438)	0.848		
Thoracic tumor chemotherapy	156	1.103 (0.756-1.609)	0.610		
Thoracic tumor surgery	156	0.991 (0.714-1.376)	0.957		

Bold values represent a valuable result.

**Table 3:** Univariate and multivariate Cox regression analyses before propensity score matching to examine the brain metastasis interval.

Characteristics	Total(N)	Univariate analysis		Multivariate analysis	
		Hazard ratio (95% CI)	P value	Hazard ratio (95% CI)	P value
pathological subtype	156	1.190 (0.853-1.660)	0.305		
age	156	1.149 (0.811-1.628)	0.434		
gender	156	0.923 (0.665-1.281)	0.631		
Whether smoke	156	0.946 (0.675-1.326)	0.748		
KPS score	156	1.680 (1.204-2.345)	**0.002**	1.226 (0.859-1.751)	0.261
Lymph node metastasis	156	1.158 (0.790-1.698)	0.452		
Whether brain metastases have occurred at the time of diagnosis	156	1997565716.821 (0.000-Inf)	0.994		
Tumor location	156	0.601 (0.393-0.919)	**0.019**	0.516 (0.331-0.802)	**0.003**
T stage	156	1.468 (1.025-2.102)	**0.036**	0.958 (0.656-1.400)	0.826
N stage	156	1.186 (0.843-1.670)	0.327		
TNM stage	156	2.952 (2.079-4.191)	**<0.001**	1.239 (0.730-2.105)	0.427
Thoracic tumor radiation	156	0.509 (0.337-0.768)	**0.001**	0.651 (0.416-1.019)	0.060
Thoracic tumor chemotherapy	156	0.439 (0.297-0.647)	**<0.001**	0.415 (0.276-0.624)	**<0.001**
Thoracic tumor surgery	156	0.212 (0.145-0.308)	**<0.001**	0.266 (0.151-0.469)	**<0.001**

Bold values represent a valuable result.

### 3.3 Survival analysis of overall survival and the brain metastasis interval after PSM

**Figure 1:**
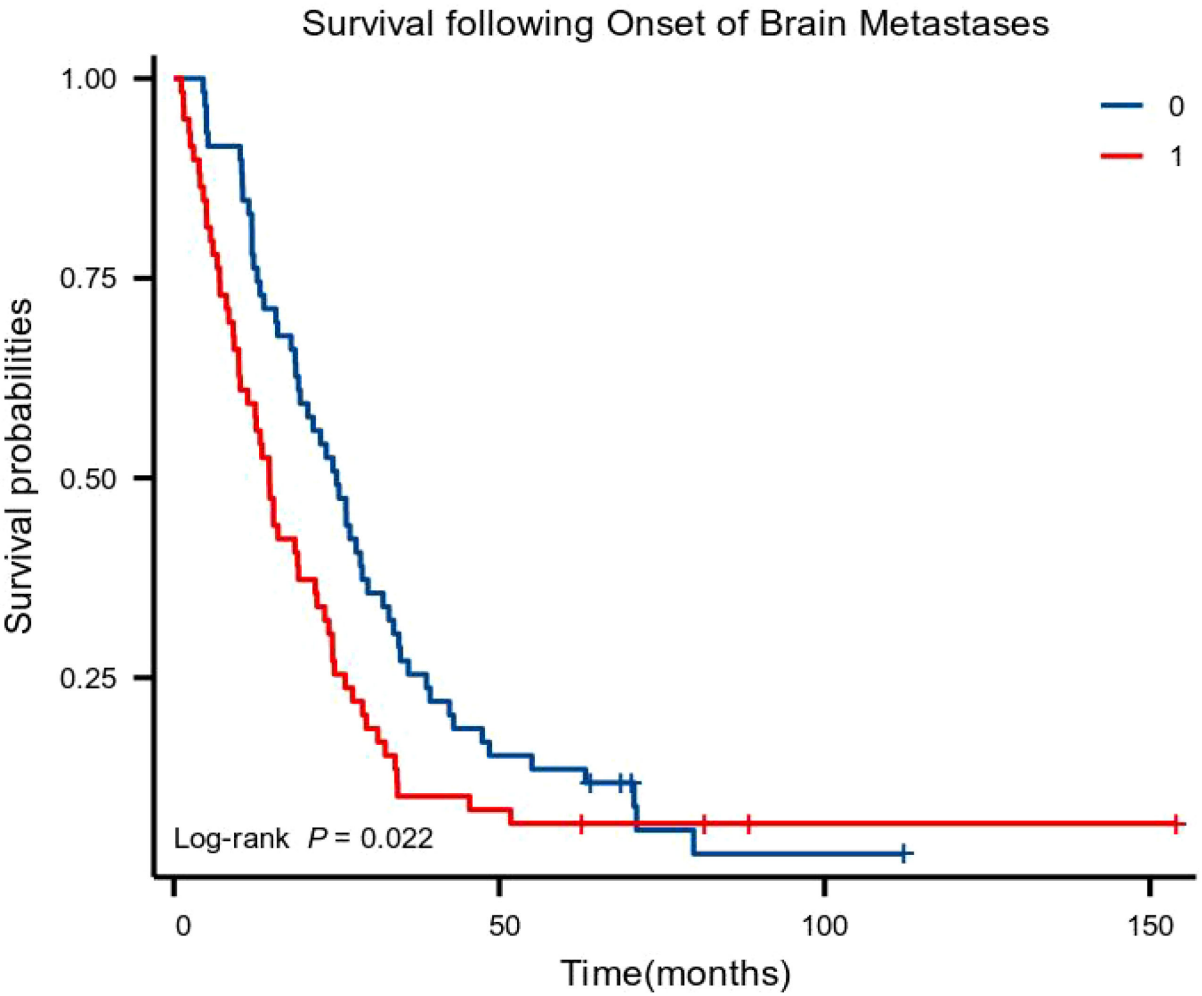
Kaplan–Meier curves for overall survival (0: moderate- to high-differentiation group; 1: poor-differentiation group).

The basic principle of PSM is to replace multiple covariates with a single score that equalizes the covariate distribution between the treatment and control groups. Before PSM, the moderate- to high-differentiation group included 89 patients, and the poor-differentiation group included 67 patients. After PSM, 59 clinically homogeneous patients were included in each group. In the moderate- to high-differentiation and poor-differentiation groups, the risk factors (age, sex, smoking status, the KPS score, lymph node metastasis, whether brain metastasis occurred at the time of diagnosis, location of the tumor in the thorax, T stage, N stage, tumor stage, chemotherapy, radiotherapy, and surgical treatment) influencing patient survival and the brain metastasis interval were balanced. Kaplan–Meier curves showed that patients with brain metastases in the moderate- to high-differentiation group had a longer overall survival (OS; P < 0.05) with a median OS (mOS) of 25.00 months (95% CI: 19.55–30.45 months) compared with an mOS of 14.67 months in the poor-differentiation group (95% CI: 11.80–17.53 months) (**Figure 1**).

In the univariate analysis, the pathological subtype was related to brain metastasis survival (HR: 1.546, 95% CI: 1.061–2.254; P = 0.023) but not to the brain metastasis interval (HR: 1.112; 95% CI: 0.762–1.621; P = 0.583). KPS, thoracic tumor staging, thoracic tumor chemotherapy, thoracic tumor radiation therapy, and thoracic tumor surgical treatment were related to the brain metastasis interval (P < 0.05). The survival of patients with brain metastases was lower in the poor-differentiation group (HR: 1.546; 95% CI: 1.061–2.254; P = 0.023) than in the moderate- to high-differentiation group in the multivariate analysis. Thoracic tumor radiation therapy (HR: 0.492; 95% CI: 0.282–0.858; P = 0.012), chemotherapy for thoracic tumors (HR: 0.399; 95% CI: 0.252–0.632; P < 0.001), and surgical treatment for thoracic tumors (HR: 0.198; 95% CI: 0.102–0.387; P < 0.001) were positive prognostic factors for the brain metastasis interval (**Tables 4**, **5**).

**Table 4:** Univariate and multivariate Cox regression analyses after propensity score matching to examine overall survival.

Characteristics	Total(N)	Univariate analysis		Multivariate analysis	
		Hazard ratio (95% CI)	P value	Hazard ratio (95% CI)	P value
pathological subtype	118	1.546 (1.061-2.254)	**0.023**	1.546 (1.061-2.254)	**0.023**
age	118	1.035 (0.701-1.528)	0.863		
gender	118	1.074 (0.734-1.572)	0.712		
Whether smoke	118	1.020 (0.698-1.491)	0.917		
KPS score	118	1.234 (0.848-1.797)	0.273		
Lymph node metastasis	118	0.986 (0.647-1.502)	0.946		
Whether brain metastases have occurred at the time of diagnosis	118	0.757 (0.490-1.170)	0.211		
Tumor location	118	0.753 (0.436-1.302)	0.310		
T stage	118	0.983 (0.660-1.466)	0.934		
N stage	118	1.124 (0.765-1.652)	0.551		
TNM stage	118	0.855 (0.587-1.244)	0.413		
Thoracic tumor radiation	118	0.839 (0.516-1.364)	0.478		
Thoracic tumor chemotherapy	118	0.971 (0.635-1.487)	0.894		
Thoracic tumor surgery	118	1.014 (0.697-1.474)	0.944		

Bold values represent a valuable result.

**Table 5:** Univariate and multivariate Cox regression analyses after propensity score matching to examine the brain metastasis interval.

Characteristics	Total(N)	Univariate analysis		Multivariate analysis	
		Hazard ratio (95% CI)	P value	Hazard ratio (95% CI)	P value
pathological subtype	118	1.112 (0.762-1.621)	0.583		
age	118	1.105 (0.746-1.637)	0.618		
gender	118	0.896 (0.610-1.314)	0.574		
Whether smoke	118	0.939 (0.641-1.376)	0.746		
KPS score	118	2.039 (1.384-3.003)	**<0.001**	1.373 (0.911-2.069)	0.130
Lymph node metastasis	118	1.000 (0.655-1.527)	1.000		
Whether brain metastases have occurred at the time of diagnosis	118	2350794642.420 (0.000-Inf)	0.995		
Tumor location	118	0.744 (0.430-1.289)	0.292		
T stage	118	1.413 (0.942-2.120)	0.095	1.031 (0.674-1.578)	0.889
N stage	118	1.131 (0.769-1.662)	0.532		
TNM stage	118	2.715 (1.822-4.046)	**<0.001**	0.852 (0.460-1.579)	0.611
Thoracic tumor radiation	118	0.443 (0.263-0.747)	**0.002**	0.492 (0.282-0.858)	**0.012**
Thoracic tumor chemotherapy	118	0.420 (0.270-0.652)	**<0.001**	0.399 (0.252-0.632)	**<0.001**
Thoracic tumor surgery	118	0.207 (0.134-0.321)	**<0.001**	0.198 (0.102-0.387)	**<0.001**

Bold values represent a valuable result.

## 4 Discussion

We evaluated the relationship between pathological subtype, patient survival after brain metastasis, and the interval between lung cancer diagnosis and brain metastases. This study revealed a longer mOS and OS in the moderate- to high-differentiation group. Furthermore, the pathological subtype of lung adenocarcinoma (P = 0.023) was revealed as an independent factor impacting survival time. Independent factors affecting the brain metastasis interval included radiation therapy for thoracic tumors (P = 0.012), chemotherapy for thoracic tumors (P < 0.001), and surgical treatment for thoracic tumors (P < 0.001).

In studies focusing on the interval between diagnosis and brain metastasis, the lung adenocarcinoma pathological subtype did not influence the brain metastasis interval; however, treatments targeting thoracic tumors (surgical treatment, chemotherapy, and radiation) tended to delay the development of brain metastases. Similarly, some studies have found that patients with lung adenocarcinoma who did not receive complementary treatments were more prone to develop brain metastasis after a definitive diagnosis of lung cancer (18, 19). However, most studies evaluating the time interval between diagnosis and brain metastasis did not group patients by pathological subtypes (no comparative data have been found at this time). Yang et al. (20) observed that shorter brain metastasis time intervals adversely affect the survival of patients undergoing surgery. Hence, our understanding of the relationship between brain metastasis time intervals and pathological subtypes needs to be improved to ensure timely treatment.

Various histological subtypes of lung adenocarcinoma show diverse clinical features, and the risk of recurrence and prognosis also differ. Yaldız et al. (21) found that solid and micropapillary histological subtypes were poor prognostic factors in invasive lung adenocarcinomas undergoing surgical treatment. Additionally, Russell et al. (22) found that micropapillary adenocarcinomas had a lower survival rate than papillary- and vesicular-dominant adenocarcinomas. Several studies exploring the relationship between pathological subtypes and brain metastasis found that patients with micropapillary and solid types are prone to brain metastases with lower survival (23–25). Consistent with previous studies, this study found a significant correlation between pathological subtypes and the progression and prognosis of brain metastases in invasive lung adenocarcinoma. Therefore, patients with a pathology suggestive of solid and micropapillary types should be closely followed up with postoperative examinations to observe tumor metastasis, and these should be treated aggressively.

The pathological subtypes also offer insights into the clinical treatment options. The response of distinct lung adenocarcinoma subtypes to targeted therapy and immunotherapy requires further investigation. For patients with invasive adenocarcinoma, there are a number of conventional targets for mutation detection related to the specific histological growth pattern of adenocarcinomas. The epidermal growth factor receptor (EGFR) gene is the most well-studied molecular target in lung cancer and is a biomarker for predicting the effectiveness of targeted therapies (26). Various studies have reported that EGFR mutations have the highest incidence rate in micropapillary tumors, followed by alveolar and solid tumor types (27–29). Studying the relationship between pathological subtype and gene mutation status provides important information to assist patients in their therapeutic choices. The expression of programmed death-ligand 1 (PD-L1) in invasive lung adenocarcinomas also varies greatly according to the histological type. PD-L1-positive tumors are more common in alveolar and solid adenocarcinomas than in other adenocarcinoma subtypes (30). This pathological classification can have a meaningful impact when screening patients for lung adenocarcinomas who are more suitable for immunotherapy, both for postoperative adjuvant chemotherapy and for treatment after recurrence. The patient’s pathological subtype may assist in selecting the most appropriate treatment regimen.

Each histological subtype of invasive lung adenocarcinoma is associated with a distinct prognosis, and the underlying mechanisms have been somewhat elucidated. One study found that, at the single-cell level, the tumor microenvironment in solid-type invasive lung adenocarcinoma was more hypoxic and acidic than that in other histological subtypes. This leads to fewer T cells, an increase in immunosuppressive myeloid cells, and a higher incidence of tumor metastasis (31). In addition, when typing lung adenocarcinomas according to different DNA methylation levels, the hypermethylated subtypes tend to be micropapillary-predominant cases. This demonstrates that patients with brain metastases from invasive pulmonary adenocarcinomas of the solid and micropapillary types have a worse prognosis, adding to the credibility of our study (32). Patients with early-stage cancer have a higher risk of metastasis if their tumors contain a highly aggressive component, requiring more stringent adjuvant therapy and close follow-up.

This study has some limitations. It was a single-center retrospective study; therefore, selection bias and confounding factors are unavoidable. Furthermore, follow-ups were not continued to obtain patient survival data. Moreover, in our cohort, the proportion of patients with gene mutations was not analyzed.

In conclusion, the findings indicate that pathological subtype is an independent risk factor affecting prognosis. Additionally, patients with poorly differentiated pathological subtypes had a lower survival rate. This provides important information for clinicians to judge patient prognosis without the need for other auxiliary techniques, as well as a reliable experimental basis for guiding the time window of treatment for this type of patient. Future prospective randomized cohort studies with large sample sizes need to be conducted for more detailed analyses. In addition, further research should consider the proportion of patients with gene mutations to draw relevant conclusions. In the near future, we expect to be able to analyze the progression of lung adenocarcinoma in terms of tumor recurrence, lymph node metastasis, and hematopoietic metastasis according to pathological subtypes, thereby leading to more accurate diagnosis and treatment.

## Data Availability

The raw data supporting the conclusions of this article will be made available by the authors, without undue reservation.
